# Podocytes Regulate Neutrophil Recruitment by Glomerular Endothelial Cells via IL-6–Mediated Crosstalk

**DOI:** 10.4049/jimmunol.1300229

**Published:** 2014-05-28

**Authors:** Sahithi J. Kuravi, Helen M. McGettrick, Simon C. Satchell, Moin A. Saleem, Lorraine Harper, Julie M. Williams, George Ed Rainger, Caroline O. S. Savage

**Affiliations:** * Centre for Translational Inflammation Research, School of Immunity and Infection, University of Birmingham, Birmingham B15 2TT, United Kingdom;; † School of Immunity and Infection, College of Medical and Dental Sciences, University of Birmingham, Birmingham B15 2TT, United Kingdom;; ‡ Academic Renal Unit, Southmead Hospital, Bristol BS10 5NB, United Kingdom;; § Wellcome Trust Clinical Research Facility, University Hospital Birmingham Foundation Trust, Birmingham B15 2TH, United Kingdom; and; ¶ Clinical and Experimental Medicine, College of Medical and Dental Sciences, University of Birmingham, Birmingham B15 2TT, United Kingdom

## Abstract

Stromal cells actively modulate the inflammatory process, in part by influencing the ability of neighboring endothelial cells to support the recruitment of circulating leukocytes. We hypothesized that podocytes influence the ability of glomerular endothelial cells (GEnCs) to recruit neutrophils during inflammation. To address this, human podocytes and human GEnCs were cultured on opposite sides of porous inserts and then treated with or without increasing concentrations of TNF-α prior to addition of neutrophils. The presence of podocytes significantly reduced neutrophil recruitment to GEnCs by up to 50% when cultures were treated with high-dose TNF-α (100 U/ml), when compared with GEnC monocultures. Importantly, this phenomenon was dependent on paracrine actions of soluble IL-6, predominantly released by podocytes. A similar response was absent when HUVECs were cocultured with podocytes, indicating a tissue-specific phenomenon. Suppressor of cytokine signaling 3 elicited the immunosuppressive actions of IL-6 in a process that disrupted the presentation of chemokines on GEnCs by altering the expression of the duffy Ag receptor for chemokines. Interestingly, suppressor of cytokine signaling 3 knockdown in GEnCs upregulated duffy Ag receptor for chemokines and CXCL5 expression, thereby restoring the neutrophil recruitment. In summary, these studies reveal that podocytes can negatively regulate neutrophil recruitment to inflamed GEnCs by modulating IL-6 signaling, identifying a potential novel anti-inflammatory role of IL-6 in renal glomeruli.

## Introduction

Recruitment of circulating leukocytes is required for immune surveillance and protective inflammatory responses to microbial infection and tissue damage. In response to inflammatory cytokines, endothelial cells (EnCs) upregulate adhesion molecules (selectins, VCAM-1, and ICAM-1), which capture flowing leukocytes. They also present chemokines and lipid signals that are necessary to stabilize adhesion and support onward migration (reviewed in Ref. 1). Deregulation of these processes is associated with the uncontrolled persistent infiltration of leukocytes into inflamed tissues that underpins chronic inflammatory diseases, as seen in vasculitic glomerulonephritis. For example, excessive neutrophil binding in the highly specialized glomerular capillary compartment is particularly evident during early nephritogenic immune responses (2–7). The mechanisms involved in neutrophil recruitment to glomerular EnCs (GEnCs) are well documented (4–10); however, much less is known about how these mechanisms are influenced by stromal cells within the underlying tissue.

We and others have previously shown that stromal cells, such as fibroblasts, tissue-resident leukocytes, or smooth muscle cells, influence the ability of neighboring EnCs to recruit leukocytes in response to cytokine stimulation (reviewed in Ref. 11). Stromal modulation of endothelial responses may occur via paracrine signaling and alteration of junctional molecule complexes (12–16). Similarly, in glomerular capillaries, neighboring glomerular epithelial cells (podocytes) may be important in regulating different physiological functions. A symbiotic paracrine communication between GEnCs and podocytes via soluble mediators is recognized during normal maintenance functions such as repair and regeneration of lost or damaged GEnCs (17–26). For example, vascular endothelial growth factor and angiopoietin-1, secreted by podocytes, bind to their cognate receptors on GEnCs and mediate crosstalk during angiogenesis (27–29). Moreover, a very recent study also highlighted the involvement of crosstalk between podocytes and endothelial cells in the production of glomerular extracellular matrix (30). The present studies focused on further identifying the intricate communication pathways between GEnCs and podocytes that regulate leukocyte recruitment and trafficking during inflammation.

IL-6 is secreted by different cells, including podocytes, and is a vital modulator of immune and inflammatory responses (31–35). IL-6 has been shown to act in a proinflammatory as well as anti-inflammatory manner, and it is thought to act as an immunological switch (36). In IL-6–deficient mice, aerosol exposure to endotoxin induced neutrophilia in the lungs and markedly higher levels of TNF-α and MIP-2 (CXCL2), suggesting that IL-6 can reduce inflammatory responses (37). IL-6 signal transduction occurs via the JAK/STAT pathway (38, 39), whereas negative regulation is mediated by the expression of suppressor of cytokine signaling (SOCS) 3 (40). Thus, the autocrine and paracrine actions of IL-6 released by podocytes in glomeruli need further investigation.

In this study, we investigated the ability to modulate the TNF-α–induced neutrophil recruitment to cocultured GEnCs using an established in vitro model system (41). We identified soluble IL-6 as key inhibitor of neutrophil recruitment in glomerular cocultures and investigated the mechanisms that are involved in resolution of inflammation in GEnC/podocyte cocultures. Understanding these pathways is of physiological relevance given the increasing availability of biological or small molecule therapeutics to modulate immune and inflammatory responses.

## Materials and Methods

### Isolation and culturing of EnCs and podocytes

Primary podocyte cultures (a gift from Prof. M.A. Saleem, University of Bristol) and primary cultures of GEnCs (Cell Systems, Kirkland, WA) were grown using EBM-2 medium (Lonza, Basel, Switzerland) as per the suppliers’ directions. Conditionally immortalized cell lines of podocytes developed from primary cultures by Prof. M.A. Saleem (42) and conditionally immortalized GEnCs developed by Dr. S.C. Satchell (43) were used in the experiments and maintained as described previously (44, 45). Cells were grown to confluence at 33°C (with 5% CO_2_) and left to differentiate at 37°C (with 5% CO_2_). Cell line podocytes differentiate in 14 d and conditionally immortalized GEnCs differentiate in 24 h. Cell lines were tested for their specific markers: GEnCs were stained for von Willebrand factor, and podocytes were stained for Wilm’s tumor-1, nephrin, and podocin (Supplemental Fig. 1). Human umbilical vein EnCs were isolated from fresh umbilical cords with ethical approval, as described previously (46).

### Neutrophil isolation

Human blood was collected from adult healthy volunteers with informed consent into 10% acid citrate dextrose anticoagulant. Neutrophils were isolated by Percoll discontinuous density gradient centrifugation (Amersham Pharmacia Biotech, Little Chalfont, U.K.) as described previously (47). Purity of neutrophils and cells were counted by microscopic inspection and using a CASY technology cell counter.

### Coculturing of EnCs on tissue culture inserts with or without being in juxtaposition to podocytes

**FIGURE 1.**
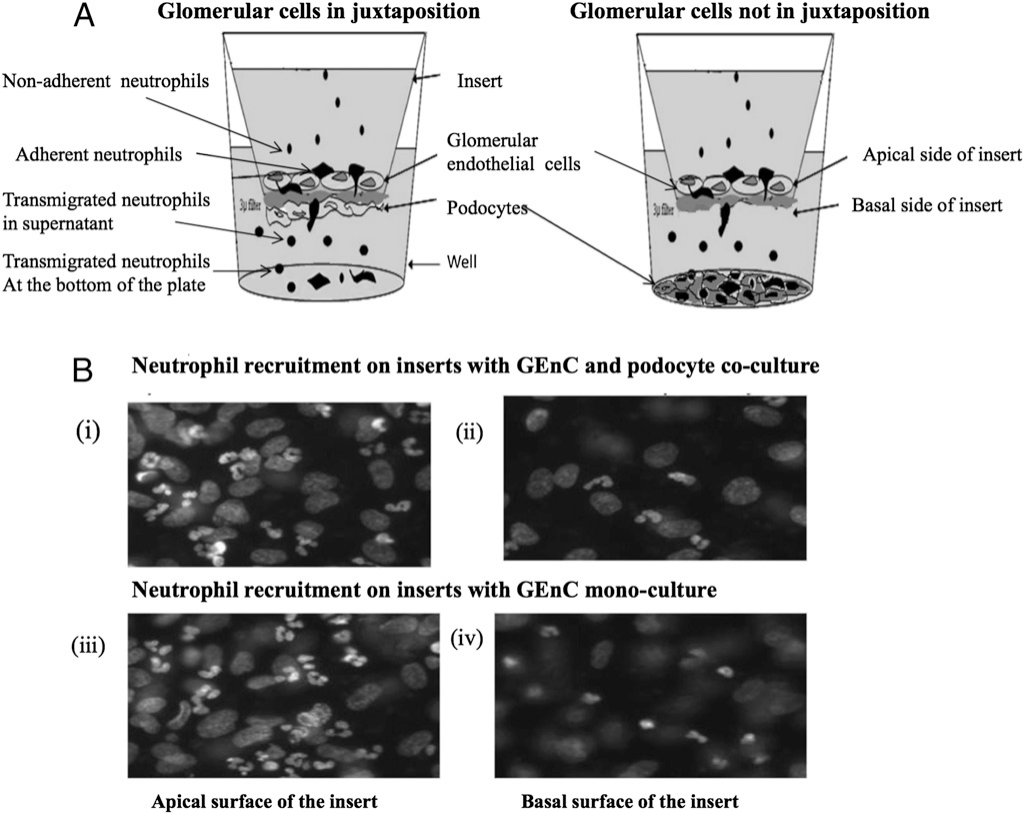
Illustration of the coculture system in the transwell. (**A**) Coculture of GEnCs and podocytes with or without being in juxtaposition. Podocytes and GEnCs on the opposite side of the porous insert formed cocultures in juxtaposition. Cocultures without being in juxtaposition were formed allowing the cell types to communicate through release of soluble mediators. Neutrophil recruitment was assessed to represent all cells that had bound to the GEnCs (i.e., firmly adherent and transmigrated). (**B**) Fluorescent micrographs showing neutrophil binding to and transmigration through GEnC/podocyte cocultures: (**i**) Apical surface of transwell insert with neutrophils adherent to GEnCs in coculture, (**ii**) basal side of transwell insert with transmigrated neutrophils adjacent to podocytes in coculture, (**iii**) apical surface of transwell insert with neutrophils adherent to GEnCs in monoculture, and (**iv**) basal side of transwell insert with transmigrated neutrophils in GEnC monocultures. Original magnification ×40.

Podocytes and EnCs (GEnCs, both primary and differentiated conditionally immortalized, and HUVECs) were harvested from confluent flasks and suspended in RPMI 1640 medium. The cells were seeded at a density of 6 × 10^4^ cells onto 3-μm porous polyethylene terephthalate transwell filter inserts (BD Pharmingen, Oxford, U.K.). Podocytes were seeded on the external surface of the transwell insert and allowed to adhere to the insert for 4–6 h at 37°C with occasional addition of medium. EnCs were seeded inside of the insert (see Fig. 1A) and allowed to settle. Light microscopy was used to determine the quality of the podocyte and EnC monolayers. This was also confirmed with bisbenizimide staining following the adhesion assay (see Fig. 1B). Monolayer integrity was assessed by measuring the electrical resistance across podocyte monolayers (ranging between 0.7 and 1.1 Ω cm^2^), GEnCs (ranging between 5.7 and 11.2 Ω cm^2^), and cocultured inserts (ranging between 10.2 and 16.3 Ω cm^2^) (*n* = 43) (Supplemental Fig. 2). The inserts were placed in 24-well companion plates after establishing the coculture and further cultured together for 24 h at 37°C (following procedures adapted from Refs. 13, 41, and 48). In further studies to highlight the importance of juxtaposition of the cell types, coculture was established without being in juxtaposition, where GEnCs were seeded inside of the insert and podocytes were seeded in the bottom of a 24-well companion plate (Fig. 1A) and conditioned for 24 h as described earlier. Parallel monocultures of EnCs and podocytes were used as comparators.

The cultures were treated with a range of TNF-α (National Institute for Biological Standards and Control, Potters Bar, U.K.) concentrations (0–100 U/ml, unless otherwise stated) 4 h prior to the addition of neutrophils. The concentration range and time period were taken from a previous study (49). Nonadherent neutrophils and transmigrated cells were collected by washing the GEnC/filter and the bottom of the well, respectively. Cells were pooled with those obtained from washes and counted using a CASY technology cell counter. The size-specific gating strategies were employed to avoid counting of nonviable podocytes or GEnCs in supernatants. Adherent neutrophils either located on the apical or basal surface of the filter or adherent to the plate were fixed, stained with 2% isotonic glutaraldehyde with 1μg/ml bisbenzimide (both from Sigma-Aldrich, Gillingham, U.K.), and analyzed using UV fluorescent microscopy and Image-Pro Plus software (Media Cybernetics, Marlow, U.K.). Using the nuclear dye bisbenzimide, the multilobed nuclei of neutrophils either on the apical or basal surface of the insert were counted. These were distinguishable from the mononuclear GEnCs or podocytes also in culture. Staining of the latter also allowed confirmation of the integrity of the GEnC and podocyte monolayers. Percentage neutrophil recruitment was determined by summing the counts from the filter, the lower supernatant and the plate (combining the adherent and total transmigrated neutrophils), in relationship to the number of neutrophils added. All data were plotted as percentage of recruited neutrophils of total added.

### Detection of cytokines and CXC chemokines

Cell culture supernatants from cocultures or monocultures were screened for cytokines and chemokines using a multiplex bead immunoassay (Invitrogen, Paisley, U.K.) and Quantikine immunoassays (R&D Systems, Abingdon, U.K.). Soluble IL-6 and CXC chemokines (CXCL8 and CXCL1) were measured using the multiplex kit according to manufacturer’s instructions, and the data were expressed in picograms per milliliter calibrated against standards.

### Cell treatments

In some experiments, GEnC monocultures were treated with 10 ng/ml IL-6 (ImmunoTools, Arlette Luttmann, Germany) for 24 h prior to TNF-α stimulation, and this was maintained throughout the experiment. Neutrophils were treated with blocking Abs against CXCR1 or CXCR2 individually and in combination (both 4 μg/ml; IgG1) (clones 501 and 19, respectively; BioSource International, Camarillo, CA) prior to their recruitment on endothelial cultures. Concentrations were followed and modified accordingly (50). To neutralize IL-6 activity, monoclonal anti–IL-6 Ab (5 μg/ml, R&D Systems, clone 6708) was added to the medium with the establishment of cocultures and maintained throughout the experiment followed by 4 h TNF-α (100 U/ml) treatment (51). In defining the molecules, which supported neutrophil migration, function-neutralizing Abs against CXC chemokines CXCL1, CXCL5, and CXCL8 were used at 2, 5, and 10 μg/ml respectively (all IgG1, clone 33160 against CXCL5, clone 31716 against CXCL1, and clone 6217 against CXCL8; R&D Systems, Abingdon, U.K.). Blocking Abs were added to the endothelium/cultures for 24 h prior to the adhesion assay. Their effects were compared with an isotype-matched mouse IgG1 mAb for all the experiments (Sigma-Aldrich, Gillingham, U.K.) as the negative control. The concentrations of the Abs had been established according to their detected levels using a multiplexing bead immunoassay.

### Knockdown of SOCS3 in GEnCs and podocytes

Cells were grown in RPMI 1640 with no antibiotics and subconfluent GEnCs, and podocytes were transfected with 25 nM/l of three target-specific commercial prevalidated SOCS3 small interfering RNAs or an appropriate scrambled control (all Santa Cruz Biotechnology and Applied Biosystems, Paisley, U.K.) or Lipofectamine alone in serum- and antibiotics-free Opti-MEM medium (Life Technologies, Paisley, U.K.). The cells were transfected using Lipofectamine (RNA iMAX, Invitrogen, Paisley, U.K.) reagent at a final concentration of 0.3% (v/v) and incubated for 48 h at 37°C in antibiotics-free Opti-MEM medium. GEnCs and podocytes with SOCS knockdown were trypsinized and either reseeded onto inserts for coculturing or used to prepare cell lysates. SOCS3 knockdown in cell lysates was measured at the mRNA level by real-time PCR and at the protein level by Western blotting to confirm knockdown. Transfected podocytes were cocultured with transfected GEnCs, as described previously, and were stimulated with TNF-α in coculture, and functional assays were performed to determine the effect of knockdown.

### Real-time PCR

For the detection of mRNAs for SOCS3, CXCL5, DARC, IL-6, and TNFR1, total cellular RNA was isolated from TNF-α (100 U/ml)–stimulated cells using an RNeasy Mini Kit 50 (Qiagen, Manchester, U.K.) following the manufacturer’s protocol, and cDNA was generated using TaqMan reverse transcription (Applied Biosystems, Carlsbad, CA). mRNA expressions were quantified by using the commercially available primers from Applied Biosystems, and the samples were amplified using the 7900 HT real-time PCR machine and analyzed using the software package SDS 2.2 (Applied Biosystems, Paisley, U.K.). Data were analyzed and expressed in comparison with GAPDH.

### Western blotting

Whole-cell lysate was subjected to 10% SDS-PAGE, transferred onto nitrocellulose membrane (GE Healthcare, Little Chalfont, U.K.), and blotted with primary Ab specific to SOCS3 (Abcam, Cambridge, U.K.). Blots were then probed with appropriate HRP-conjugated anti rabbit secondary Ab (Cell Signaling Technology, Hitchin, U.K.). Immunodetection was carried out using the ECL Plus kit (GE Healthcare Life Sciences, Amersham Place, U.K.) followed by exposure to x-ray film for 15 min.

### Statistical analysis

Results are given as a percentage of recruitment neutrophils from the total neutrophils added to the system. Data were assessed as being parametric or nonparametric prior to the use of an appropriate statistical test. Variation between multiple treatments was evaluated using an ANOVA or a Friedman test, followed by Bonferroni or Dunnett post hoc multiple comparison tests as appropriate. Variations were also analyzed by a paired *t* test, an unpaired *t* test, and a Mann–Whitney *U* test. A *p* value <0.05 was considered significant. All the data were analyzed using Prism (GraphPad Prism Software, San Diego, CA).

## Results

### The effect of podocytes on the recruitment of neutrophils to cocultured endothelial cells

**FIGURE 2.**
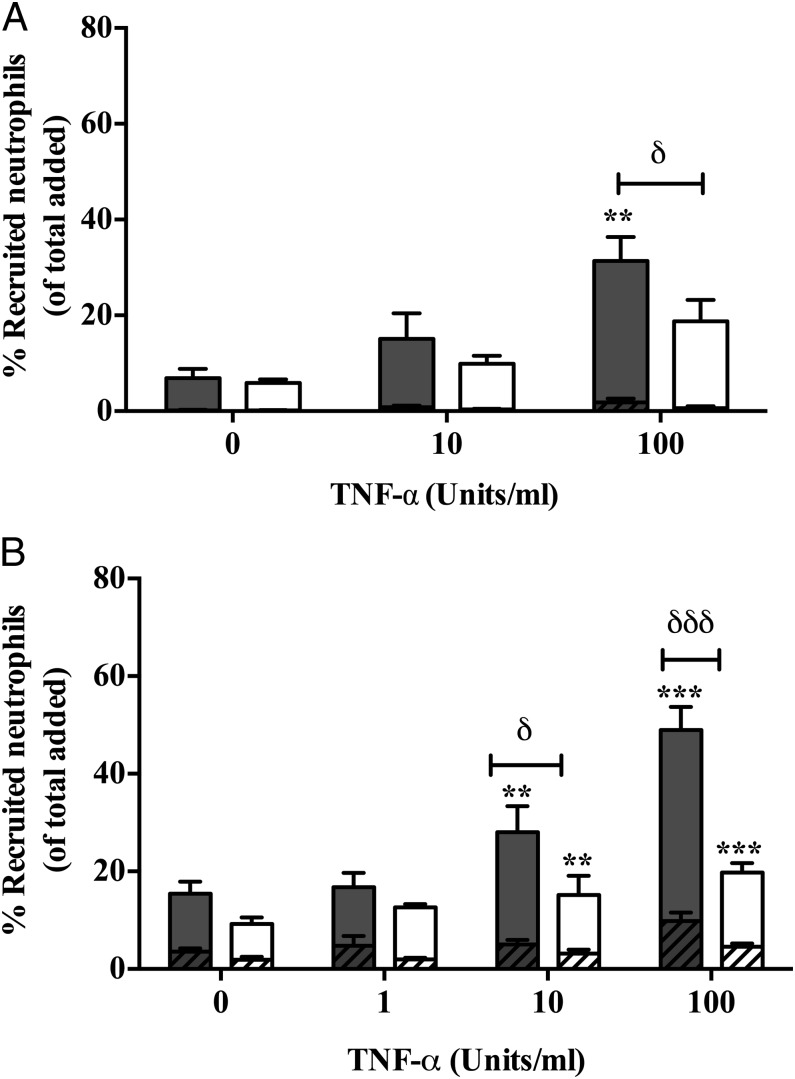
Recruitment of neutrophil to TNF-α–treated GEnC mono- and cocultures. (**A**) Primary or (**B**) immortalized cell line cultures of GEnCs and podocytes were cultured in juxtaposition (open bars) and stimulated with TNF-α (0-100 U/ml). GEnC monocultures (filled bars) were assessed as controls. Neutrophils were allowed to adhere and migrate through the constructs. Neutrophil recruitment was assessed at 1 h and taken to represent all cells that had bound to the GEnCs (i.e., firmly adherent [hashed bars] and transmigrated [plain bars]). Data are means ± SEM. (A) *n* = 4 and (B) *n* = 11–32. **p < 0.01, ***p < 0.0001 for the effect of cytokine treatment as determined by a paired Wilcoxon signed-rank test between treatments within cultures. ^δ^*p* < 0.05, ^δδδ^*p* < 0.0001 for difference between cocultures and monocultures as determined by a Mann–Whitney *U* test.

The effect of podocytes on the ability of endothelium to support neutrophil recruitment was examined in the presence of an inflammatory stimulus. In a static adhesion assay, GEnCs were cultured adjacent to podocytes (either primary or conditionally immortalized) on apical and basal surfaces of a porous filter in juxtaposition for 24 h (Fig. 1). Cocultures supported neutrophil recruitment in a TNF-α dose–dependent manner, with most recruited cells undergoing transendothelial migration (Fig. 2). Importantly, the level of neutrophil recruitment by GEnCs cocultured in juxtaposition to podocytes was significantly lower (by up to 50%) at 100 U/ml TNF-α (high dose) when compared with GEnC monocultures (Fig. 2). Similar observations were made using primary (Fig. 2A) and immortalized cells (both endothelial and podocytes) in coculture (Fig. 2B). All further experiments were performed using immortalized cell lines, as these could support the inhibitory effects of coculture and were considerably easier to acquire and coculture.

In comparison, when HUVECs were cocultured with podocytes, the reduction in neutrophil recruitment was not observed (Table I). Thus, podocytes appear to regulate inflammatory responses mediated by GEnCs in a tissue-specific manner.

**Table I.** Neutrophil recruitment to HUVECs cultured alone or with and without being in juxtaposition to podocytes

	TNF-α Stimulation (U/ml)		
	0	10	100
HUVECs in monocultures	4.9 ± 1.5	15.7 ± 0.8*	24.57 ± 3.01**
HUVECs in direct contact with podocytes	11.43 ± 4.7	21.53 ± 5.7	38.7 ± 9.8*
HUVECs with no direct contact	5.7 ± 1.13	14.5 ± 1.9*	24.01 ± 1.6**

HUVECs and podocytes were seeded in juxtaposition on opposite sides of a porous insert, prior to stimulation with increasing concentrations of TNF-α (0–100 U/ml). HUVEC monocultures were assessed as controls. Neutrophils were allowed to adhere and migrate through the constructs. Neutrophil recruitment was assessed at 1 h and taken to represent all cells that had bound to the HUVECs (i.e., firmly adherent and transmigrated). Data are means ± SEM. *n* = 3 expressed as percentage of neutrophil recruitment of total added neutrophils.

Friedman test shows a significant effect of cytokine treatments on neutrophil recruitment (*p* < 0.001), *p < 0.05, **p < 0.001, compared with untreated within individual cultures by a Dunnett post hoc test.

### Role of soluble mediators released in cocultures on neutrophil recruitment

**FIGURE 3.**
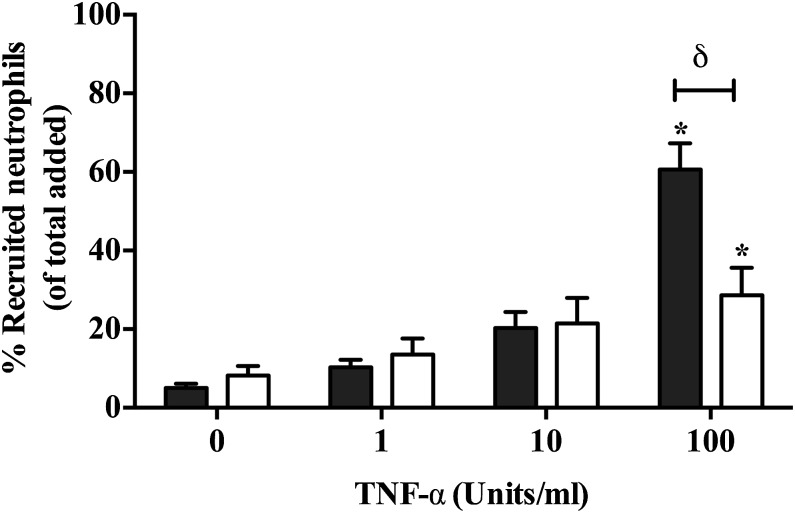
Role of soluble mediators in regulating neutrophil recruitment to GEnCs. GEnCs were seeded onto the apical surface (i.e., inside) a porous filter. Podocytes were seeded onto the bottom of the well. Cocultures were formed by placing the GEnC-coated filter above the podocyte-coated well, allowing the cell types to communicate through release of soluble mediators. Cytokine-induced neutrophil recruitment was assessed at 1 h. Data are means ± SEM (*n* = 4). A Friedman test shows a significant effect of cytokine treatments on neutrophil recruitment (*p* < 0.001). *p < 0.05 comparing the effects of TNF-α treatment to untreated cultures by a Dunnett post hoc test, ^δ^*p* < 0.05 comparing cocultures (open bars representing cocultures) to monocultures (filled bars) by an unpaired *t* test.

Next we investigated the role of soluble mediators in regulating the inflammatory response of cocultured GEnCs. To address this, we cultured GEnCs on a transwell filter above podocytes cultured in the bottom of the culture dish, thus separating each cell type (Fig. 1B). In this format, neutrophil recruitment was significantly reduced compared with a monoculture of GEnCs (Fig. 3), indicating that the altered neutrophil recruitment was at least partially dependent on a soluble mediator. As expected, when HUVECs were cultured in this format there was no significant alteration in the behavior of neutrophils (Table I).

### IL-6 mediates the anti-inflammatory crosstalk between podocytes and GEnCs

**FIGURE 4.**
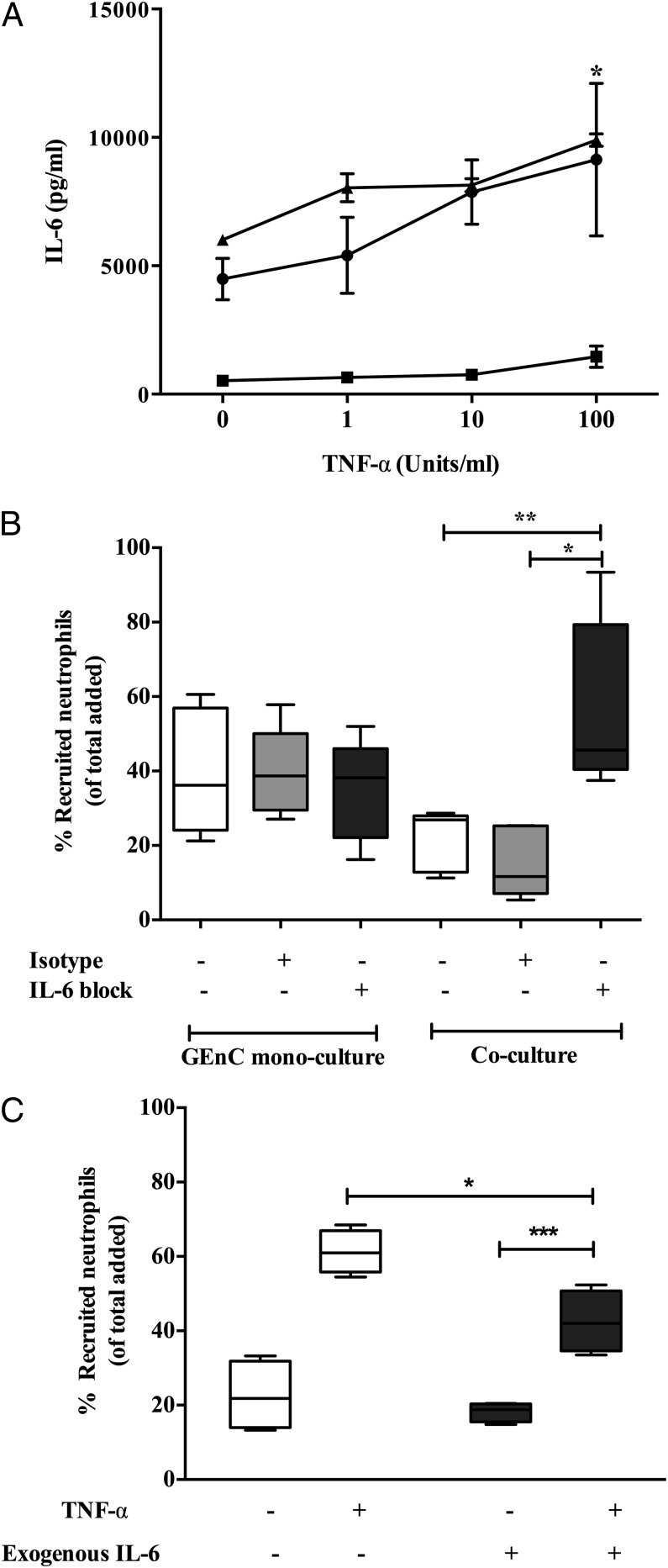
Role for IL-6 in negative regulation of neutrophil recruitment in cocultures. (**A**) Detection of IL-6 in supernatants of GEnC and podocyte cocultures (▴) and monocultures of GEnC (▪) and podocytes (●) that were treated with and without TNF-α. Data are means ± SEM (*n* = 4). Two-way ANOVA shows a significant effect of cytokine treatment on IL-6 production (*p* < 0.05). *p < 0.05 comparing IL-6 production between cocultures and GEnC monocultures by a Bonferroni post hoc test. (**B**) Neutralization of IL-6 in cultures by incorporation of 5 μg/ml anti–IL-6 Ab (labeled as IL-6 block) into TNF-α (100 U/ml)–stimulated cultures for 24 h starting from the time of establishing the cocultures. Neutrophil recruitment was assessed at 1 h and is expressed as percentage of cells recruited. Data are represented as minimum to maximum levels of recruitment (*n* = 5). The effects were compared with an isotype-matched mouse IgG in these experiments. *p < 0.05, **p < 0.001 comparing the effect of IL-6 neutralization with controls by a paired *t* test. (**C**) Recapitulation of coculture by addition of exogenous IL-6 in GEnC monocultures (*n* = 4). *p < 0.05 by Mann–Whitney *U* test when comparing treatments, ***p < 0.0001 comparing the effect of cytokine treatment by a paired Wilcoxon signed-rank test between treatments within cultures, ***p < 0.0001 comparing the effect of cytokine treatment by a paired Wilcoxon signed rank test between treatments within cultures.

To identify the specific soluble mediators responsible for the inhibition in neutrophil recruitment we compared supernatants generated from GEnCs under conditions of monoculture or during coculture with podocytes. Multiplex ELISA showed low levels of IL-6 in supernatants from GEnC monocultures treated with or without exogenous TNF-α (Fig. 4A). Alternatively, TNF-α–stimulated podocyte monocultures and GEnC/podocyte cocultures released significantly higher levels of IL-6 compared with unstimulated cultures (Fig. 4A). In contrast, HUVECs suppressed podocyte production of IL-6 during coculture (Supplemental Fig. 3), possibly explaining why we observed no inhibition of neutrophil recruitment in these constructs. These data strongly suggests that IL-6 generated in GEnCs/podocytes in cocultures was primarily released by podocytes.

We have previously reported that IL-6 produced during endothelial–dermal fibroblast coculture suppresses cytokine-induced lymphocyte recruitment (51). Thus, we considered whether IL-6 may be playing a similar role in the present study. When the biological activity of IL-6 was removed from coculture supernatants using a function neutralizing Ab, the inhibitory effects of coculture were lost (Fig. 4B). Moreover, treating GEnC monocultures with exogenous IL-6 (10 ng/ml, equivalent to the levels released in coculture) significantly reduced neutrophil recruitment (Fig. 4C). These results demonstrate that IL-6 is key mediator in the anti-inflammatory crosstalk that regulates neutrophil recruitment in cocultures of GEnCs and podocytes.

### SOCS3 is important for IL-6–mediated regulation of neutrophil recruitment in cocultures

**FIGURE 5.**
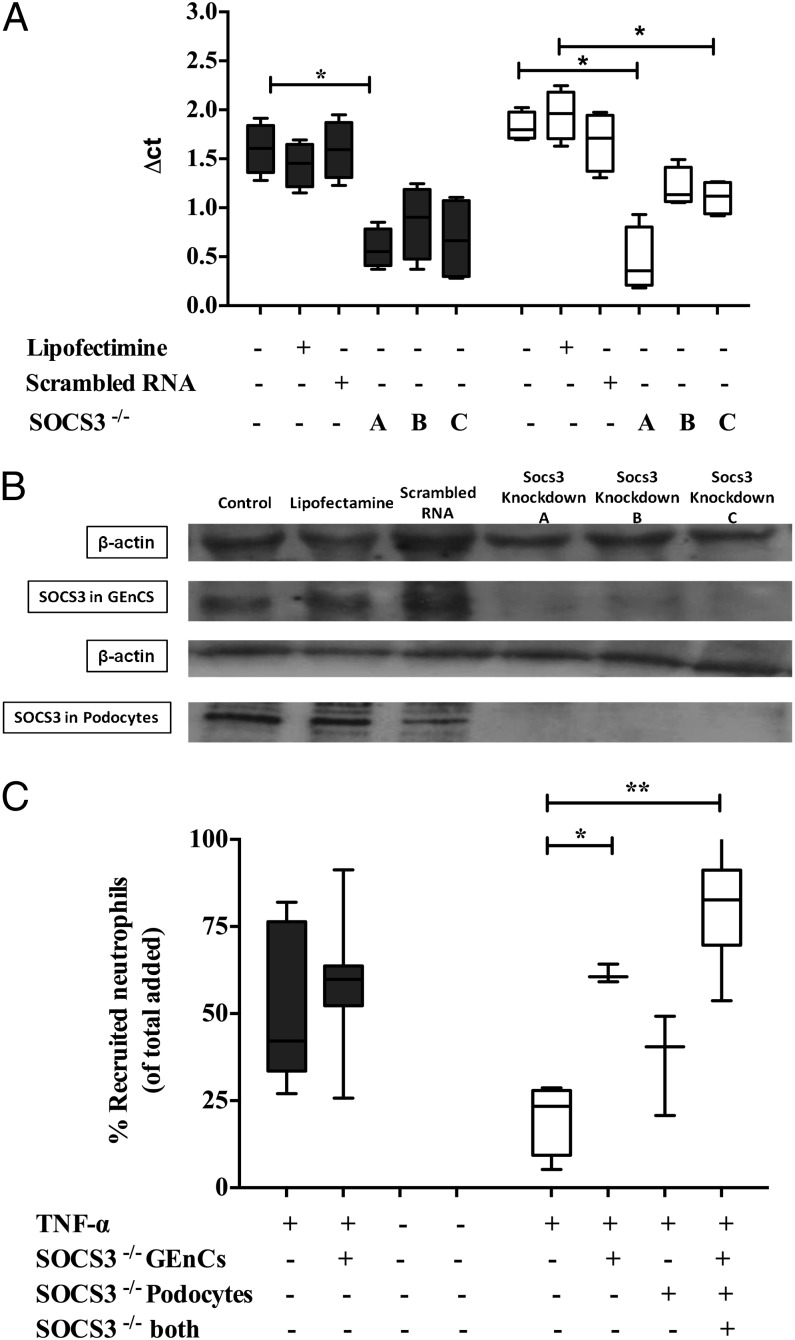
SOCS3-mediated downregulation of neutrophil recruitment in cocultures of GEnCs and podocytes. (**A**) Detection of SOCS3 mRNA in TNF-α (100 U/ml)–stimulated cultures of GEnCs (filled bars) and podocytes (open bars) by quantitative PCR following SOCS3 knockdown with three different oligomers (labeled A [mixture of three oligomers] and B and C [individual oligomers]) or a scrambled control. Lipofectamine presence is indicated, whereas “untreated” indicates absence of Lipofectamine, scrambled control, or SOCS oligomers. Data are minimum to maximum (*n* = 3–6). A Friedman test compared the effect of SOCS3 knockdown (*p* < 0.05). *p < 0.05 comparing controls within cultures by a Dunnett post hoc test. (**B**) Western blotting confirming the SOCS3 protein knockdown in GEnCs and podocytes compared with controls (untreated cultures, Lipofectimine-treated cultures, and scrambled RNA-treated cultures). β-actin was used as loading control. (**C**) Neutrophil recruitment in cocultures (open bars) and GEnC monoculture (filled bars) with SOCS3 knockdown (−/−) with three different oligomers (labeled A [mixture of three oligomers] and B and C [individual oligomers]) or a scrambled control. Lipofectamine presence is indicated, whereas “untreated” indicates absence of Lipofectamine, scrambled control, or SOCS oligomers. Neutrophil recruitment in cocultures was also tested with SOCS3 knockdown in only one cell type. Neutrophil recruitment was assessed in TNF-α (100 U/ml)–treated cultures at 1 h. Data are minimum to maximum of recruited cells (*n* = 3–7). A Friedman test compared the effect of SOCS3 knockdown (*p* < 0.05). *p < 0.05, **p < 0.01 comparing with controls by a Dunnett post hoc test.

SOCS3 belongs to a family of cytokine-inducible SH2 proteins that are shown to be involved in the negative regulation of cytokine signaling involving JAKs and STAT activity. Importantly, SOCS3 is known to be involved in blocking the downstream signaling from the IL-6 receptor (reviewed in Ref. 52). In this study, we used three small interfering RNA oligomers in combination or individually to knockout SOCS3 expression in both GEnCs and podocytes prior to coculture. Successful knockdown of SOCS3 was confirmed by quantitative real-time PCR analysis (Fig. 5A) and Western blotting (Fig. 5B), and it was unaffected by the presence of scrambled control. Inhibition of SOCS3 expression in podocytes and GEnCs simultaneously or GEnCs alone in coculture released neutrophils from the inhibitory effects of coculture (Fig. 5C). Interestingly, however, the loss of SOCS3 in podocytes alone had no significant effect on neutrophil recruitment to the cocultured cells. These observations indicated that SOCS3 signaling in GEnCs is crucial in cocultures and that IL-6 may regulate the function of both cell types simultaneously.

### The role of CXC chemokines and their receptors in neutrophil recruitment to cocultures

**FIGURE 6.**
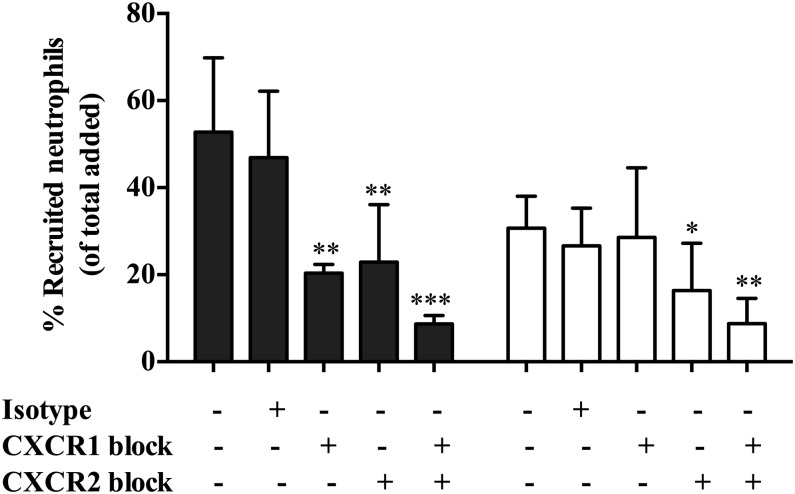
Role of CXC receptors in supporting neutrophil migration. CXCR1 and CXCR2 receptors were blocked either individually or in combination using respective blocking Abs (4 μg/ml) on neutrophils for 15 min prior to addition in both GEnC monocultures (filled bars) and cocultures (open bars) stimulated with TNF-α (100 U/ml). Neutrophil recruitment was assessed at 1 h and expressed as percentage of added cells. The effects were also compared with an isotype-matched mouse IgG in these experiments. Data are means ± SEM (*n* = 5). ANOVA shows a significant effect of treatment on neutrophil migration (*p* < 0.05). *p < 0.05, **p < 0.01, ***p < 0.0001 comparing the effect of receptor blocking on neutrophil migration with their respective controls within cultures by a Bonferroni post hoc test. Untreated, absence of isotype control Ab, CXCR1, and anti-CXCR2 blocking Abs.

The CXCR1, CXCR2, and their CXC chemokine ligands are recognized as key mediators in neutrophil recruitment (53). In the present study, we performed blocking studies to elucidate the role of these molecules in our own assays. In agreement with previous publications (50), both CXCR1 and CXCR2 played a role in supporting neutrophil migration across TNF-α–treated GEnC monocultures (Fig. 6). Importantly, however, only CXCR2 interactions were required for neutrophil recruitment to coculture GEnCs (Fig. 6). Blocking both receptors in combination further reduced neutrophil migration in both mono- and cocultures (to <5%) when compared with blocking each receptor individually (Fig. 6).

**FIGURE 7.**
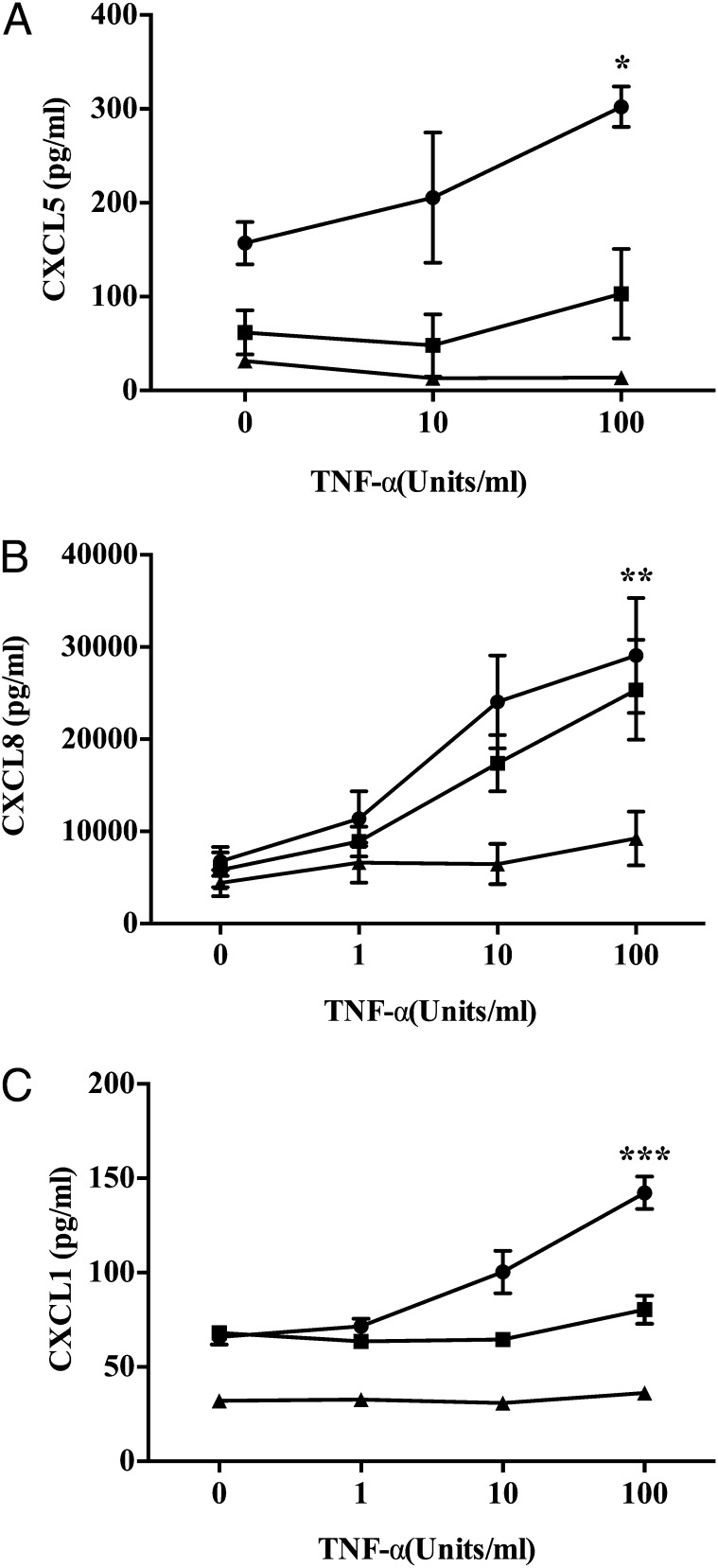
Detection of CXCL1, CXCL5, and CXCL8 in cocultures and monocultures. Detection of soluble (**A**) CXCL1, (**B**) CXCL5, and (**C**) CXCL8 in supernatants collected from TNF-α–stimulated cocultures (●), GEnC monocultures (▪), and podocyte monocultures (▴). Data expressed as means ± SEM (*n* = 3). *p < 0.05, **p < 0.01, ***p < 0.0001 comparing the difference in chemokine expression between cocultures and GEnC monocultures by two-way ANOVA.

**FIGURE 8.**
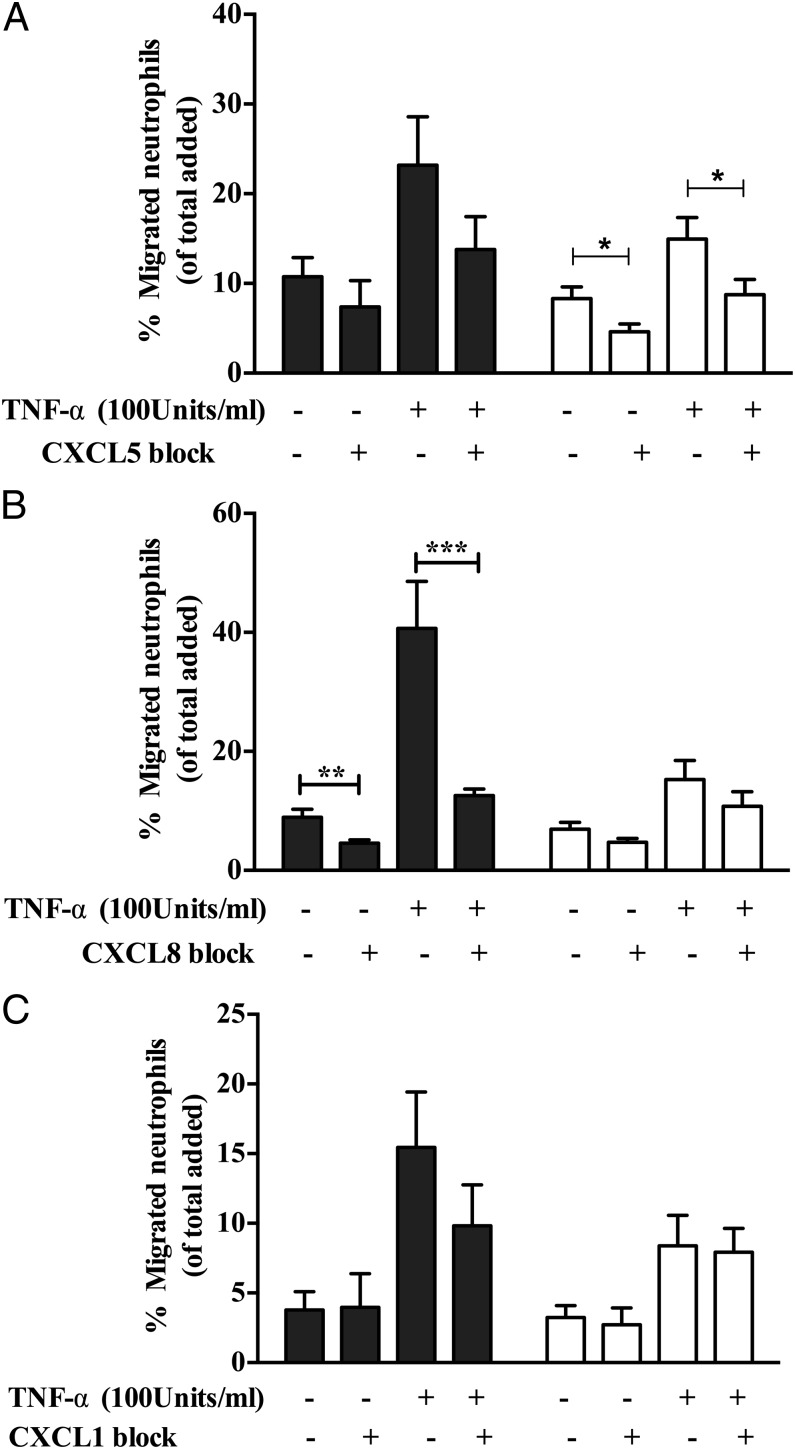
Neutralization of CXCL1, CXCL5, and CXCL8 in cocultures and monocultures. Cultures were treated with function neutralizing Abs against (**A**) CXCL1 (2 μg/ml), (**B**) CXCL5 (5 μg/ml), and (**C**) CXCL8 (10 μg/ml) for 24 h prior to addition of neutrophils. Data are means ± SEM (*n* = 5–6). *p < 0.05, **p < 0.01, ***p < 0.0001 comparing the effect of blocking chemokine function in cultures with their controls by a paired *t* test.

To confirm which chemokines were involved, we examined the expression of the CXCL5 (epithelial neutrophil–activating peptide 78), CXCL8 (IL-8), and CXCL1 (growth-related oncogene α) in culture supernatants. There was a significant increase in the levels of all analytes in coculture supernatants compared with GEnCs in monoculture (Fig. 7). Thus, to determine which of these were functionally relevant we blocked their activity with neutralizing Abs. Importantly, only CXCL5 blockade significantly reduced neutrophil recruitment to cocultured GEnCs (Fig. 8). Thus, in this coculture model of the human, glomerulus neutrophil trafficking appears to be regulated through the activation of CXCR2 by CXCL5. Interestingly, this is a pattern of activity we have observed in another assay system using endothelial cells cocultured with fibroblasts from the rheumatoid joint. In the arthritis model CXCL5 was preferentially deposited on the EnC surface by the action of the DARC (54), even though other CXC ligands were present at greater concentrations.

### SOCS3 knockdown upregulates the expression of inflammatory genes

**FIGURE 9.**
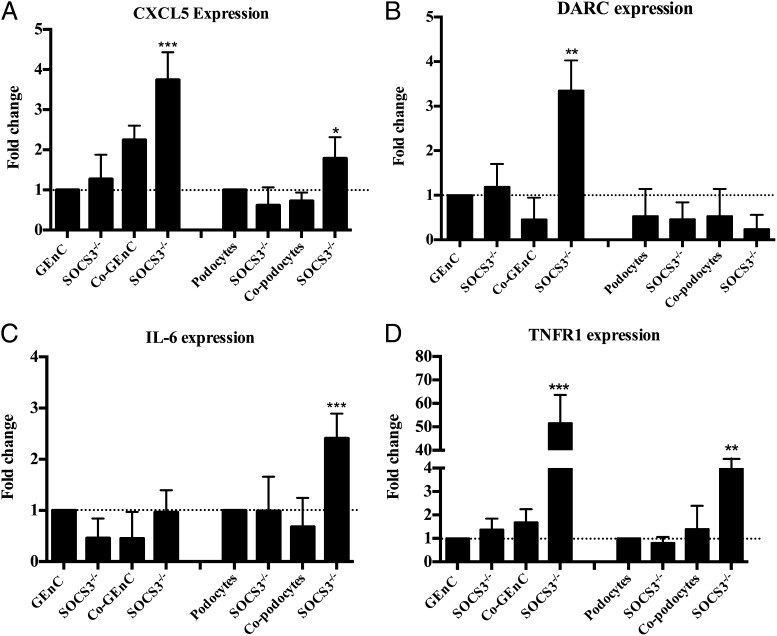
Effect of SOCS3 deletion in the upregulation of genes that support neutrophil recruitment in cocultures. Quantification of expression of genes (**A**) CXCL5, (**B**) DARC, (**C**) IL-6, and (**D**) TNFR1 that supported the neutrophil recruitment in cocultures, prior to and after depletion of SOCS3, in GEnCs and podocytes from mono- and cocultures compared with monocultures without SOCS3 knockdown. Data are expressed as fold change to TNF-α–stimulated monocultures (*n* = 5). *p < 0.05, **p < 0.01, ***p < 0.0001 comparing the difference in gene expression with the stimulated monocultures without SOCS3 knockdown by an unpaired *t* test.

Because deletion of SOCS3 has been shown to prolong activation of STAT1 and STAT3 following IL-6 stimulation, thereby enhancing inflammatory responses (54–56), we wondered whether a similar mechanism was operating in our cocultures. Specifically, we were interested in changes in the key regulators of neutrophil trafficking that we had identified above, and thus we examined the gene expression of CXCL1, CXCL5, CXL8, DARC, and TNFR1 in GEnCs or podocytes before and after SOCS3 knockdown. In the absence of SOCS3, CXCL5 and DARC were upregulated in GEnCs from cocultures (Fig. 9A and 9B, respectively), whereas CXCL1 and CXCL8 expression were unaffected (data not shown). Furthermore, we also detected upregulated expression of IL-6 in podocytes from cocultures and TNFR expression in both GEnCs and podocytes from cocultures upon knockdown of SOCS3 (Fig. 9C and 9D, respectively). Taken together, these results imply that IL-6–induced SOCS3 signaling has anti-inflammatory effects by downregulating the expression of TNFR on both of the cocultured cells, but also by reducing the expression of the CXC chemokine (CXCL5) despite it being the dominant chemokine released in supernatants from cocultures, and it thus regulates neutrophil trafficking. Additionally, reducing the expression of DARC may decrease the efficiency with which CXCL5 is translocated and expressed on the surface of cocultured endothelial cells, enabling proper presentation of CXCL5 on the surface of GEnCs in cocultures. These results also support the previous evidence that SOCS3 negatively regulates IL-6 signaling and coordinates appropriate cytokine-mediated biological responses, and they suggest a unique regulatory mechanism to control neutrophil recruitment in glomeruli under homeostasis.

## Discussion

In glomeruli, the maintenance of vascular endothelial functions depends on the crosstalk with closely apposed podocytes and GEnCs. The purpose of this study was to reconstruct the glomerular environment to determine whether such crosstalk was an important arbiter of the recruitment of neutrophils during inflammation.

The processes of leukocyte recruitment (adhesion and migration) have been elucidated in vitro using isolated leukocytes and cultured EnCs, with supporting evidence from intravital models of inflammation (55–58). However, in vivo validation has been important, because the behavior of EnCs is influenced by stromal cells and soluble mediators released in their local environment. Thus, to reflect the in vivo situation more precisely in vitro, it is necessary to develop integrated multicellular models that better reflect the inflammatory environment. In the present study we developed an experimental model of the glomerulus to mimic the context of acute inflammation in a tissue-specific manner. Monolayers of GEnCs and podocytes were cultured together, separated by a semipermeable membrane, allowing us to study how the interactions between these two cell types influenced the recruitment of neutrophils during inflammation.

A key observation was that the presence of podocytes significantly reduced the ability of inflamed endothelium to support neutrophil recruitment. This regulatory phenomenon was observed with varying potency across a range of TNF-α concentrations, indicating that podocytes are capable of modifying the responses of GEnCs to TNF-α. Podocytes exerted their anti-inflammatory effects both in the presence and absence of close proximity with the endothelium, strongly suggesting the influence of a soluble mediator. Moreover, TNF-α upregulated IL-6 release by podocytes in coculture with GEnCs, and neutralizing IL-6 reversed the observed reduction of neutrophil binding. Importantly, addition of exogenous IL-6 to GEnC monocultures recapitulated the reduction in neutrophil recruitment observed in cocultures. This demonstrates that IL-6 can act in an anti-inflammatory manner to regulate neutrophil recruitment in the glomerular environment. Interestingly, HUVECs cocultured with podocytes were unable to suppress neutrophil binding. Indeed, HUVECs downregulated IL-6 release in podocytes. Collectively, these observations indicate that podocytes communicate with GEnCs in a tissue-specific manner through the medium of IL-6 secretion and are capable of influencing endothelial responses through a range of inflammatory states.

Podocytes are a known source of IL-6 that could play a critical role in modulation of progressive glomerular disease (31, 37, 59). Previous in vivo and in vitro studies have demonstrated the role for IL-6 in attenuation of LPS-induced effects on neutrophil infiltration, and IL-6 also suppressed the response of inflammatory cytokines in a number of models (37, 40, 60–62). Interestingly, these models exhibited reduced, as well as enhanced, leukocyte adhesion to EnCs depending on the nature of the stromal cells with which they were cocultured (12, 51).

The later part of our study establishes the mechanisms underpinning the IL-6–mediated inhibition of TNF-α–mediated inflammatory responses in cocultures. Our observations demonstrate an upregulation of SOCS3, which obstructed the IL-6 signaling pathway, and we further demonstrated that SOCS3 depletion in both GEnCs and podocytes or GEnCs alone in cocultures resulted in increased neutrophil recruitment in TNF-α–stimulated cocultures. Indeed, previous studies have demonstrated a role for SOCS3 in human IL-6–mediated regulation of neutrophil adhesion (63). IL-6 can induce rapid induction of SOCS gene transcription, including SOCS3, suggesting a role for SOCS in a negative feedback loop regulating IL-6 signal transduction. Moreover, SOCS3 deficiency can have deleterious effects allowing prolonged activation of STAT1 and STAT3 after IL-6 stimulation, and it was shown to promote the expression of IFN-inducible genes (64–66). Loss of SOCS3 activity also led to enhanced production of G-CSF and IL-6 in a mouse model of inflammatory arthritis (67, 68). Similarly, our studies further demonstrate that SOCS3-deleted cells from cocultures show upregulated expression of inflammatory genes such as TNFR1 and IL-6 in podocytes and elevated levels of CXCL5 and TNFR1 expression in GEnCs that supported increased neutrophil recruitment in SOCS3 knocked down cocultures. Similar mechanisms could be involved in GEnC with exogenous IL-6 in cultures; however, these mechanisms have not yet been identified. Collectively, available evidence suggests that upregulation of SOCS3 by IL-6 can suppress IL-6 signaling via negative feedback loops and that deletion of SOCS3 upregulates the expression of inflammatory genes in the GEnC and podocyte cocultures.

Furthermore, the mechanism involved in efficient migration of minimally recruited neutrophils in cocultures was analyzed. The residual neutrophil recruitment to the cocultures appeared to be more efficient at migrating through the membrane, indicating a potential role for chemokines and their receptors. Neutrophil chemokine receptors (CXCR1 and CXCR2) were shown to have key role in transmigration across TNF-α–treated GEnC monocultures, in agreement with previous findings (50). Interestingly, in cocultures, blockade of CXCR2 on neutrophils was sufficient to reduce the residual neutrophil migration in cocultures, and double blocking strongly indicates the nonredundant role for CXC receptors in supporting the neutrophil migration in our cultures. The CXCR2 role in cocultures suggests the possible involvement of chemokines other than CXCL8, because CXCR2 binds multiple ELR chemokines (44, 45, 69). Importantly, CXCR1 binds to ligands CXCL8 and CXCL6 with high affinity, whereas CXCR2 is more promiscuous and binds CXCL1, CXCL2, CXCL3, CXCL5, CXCL6, CXCL7, and CXCL8 (44, 45, 69). Supernatant analysis demonstrated a prominent increase in soluble CXCL1, CXCL5, and CXCL8 in monocultures and cocultures, and blocking these molecules on GEnCs revealed a role for these chemokines, to varying degrees, in supporting neutrophil recruitment in glomerular cocultures. In GEnC/podocyte cocultures, CXCL5–CXCR2 interactions seem to be playing a dominant role, because blocking CXCL5 activity but not CXCL8 or CXCL1 significantly reduced the residual neutrophil recruitment in cocultures. This suggests selective presentation of CXCL5 on GEnC surfaces in cocultures. In this regard, previous studies using multicellular constructs from our group and others have reported that DARC presents CXCL5 on the endothelial surface, triggering integrin activation and stabilization of adherent leukocytes (14, 54, 70). Moreover, the level of DARC expression on the endothelium has been shown to facilitate chemokine-mediated leukocyte trafficking (70, 71). In the present study, we observed a SOCS3-dependent reduction in DARC gene expression in cocultured GEnCs. Appropriate chemokine presentation is required for stabilizing neutrophil adhesion, and in further support of endothelial transmigration, a deficiency in this process accounted for the reduction in neutrophil recruitment in our coculture. Furthermore, CXCL5 binding to DARC has been reported to regulate the availability of other chemoattractants such as CXCL1 and CXCL2 (71, 72). DARC knockout mice were protected from postischemic acute renal failure owing to impaired renal neutrophil recruitment and disruption in endothelial chemokine presentation (73–75). Importantly, specificity of chemokines is involved in IL-6–mediated signaling, an observation that is analogous to our findings in a coculture model of the glomerulus.

Interestingly, the presently reported reduction of neutrophil recruitment may be specific to GEnCs because the podocyte-mediated reduction of neutrophil recruitment was not observed in the cocultures of HUVECs and podocytes. The regulation of neutrophil recruitment may have been absent from HUVEC cocultures owing to lower levels of IL-6 release (Supplemental Fig. 3), suggesting that HUVECs may actually downregulate IL-6 production from podocytes. Our previous studies have shown that exogenous IL-6 can reduce the ability of HUVECs to respond tocytokine stimulation as measured by their ability to capture lymphocytes (51) or neutrophils from flow (76). Moreover, addition of soluble IL-6Rα in conjunction with IL-6 has been shown to be required to induce adhesion of neutrophils to EnCs (77).

Thus, IL-6–dependent cellular crosstalk, capable of modulating endothelial activation and leukocyte adhesion, may be defined by the exact milieu (41). However, the balance shifts with magnitude and duration of response, leading to inflammation and disease, as seen in rheumatoid arthritis (78), and conceivably this balance could also change unfavorably during some glomerular diseases.

In conclusion, the present studies reveal that podocytes play a role in the modulation of neutrophil recruitment to GEnCs by releasing IL-6, which, in turn, initiates negative feedback loops with upregulation of SOCS3. Although SOCS3 seems to be the dominant molecule in this podocyte-mediated regulatory process, the subsequent anti-inflammatory pathways have not been defined in this model, and by analogy with other systems, inhibition of STAT signaling may occur. These observations highlight the anti-inflammatory paracrine and autocrine actions of podocyte-derived IL-6. It is conceivable that these favorable effects of IL-6 may be modified during severe or prolonged episodes of glomerular inflammation. Additionally, the present observations add a note of caution regarding the possibility of worsening renal inflammation by therapies that target IL-6.

## Supplementary Material

Data Supplement
