# Supplementary material for: Podocytes Regulate Neutrophil Recruitment by Glomerular Endothelial Cells via IL-6–Mediated Crosstalk
Source: J Immunol. 2014 May 28;193(1):234–43. doi: 10.4049/jimmunol.1300229 (PMC4067868; doi:10.4049/jimmunol.1300229)
Supplement: Data Supplement [file supp_193_1_234__index.html]

Podocytes Regulate Neutrophil Recruitment by Glomerular Endothelial Cells via IL-6–Mediated Crosstalk — Data Supplement 

# Podocytes Regulate Neutrophil Recruitment by Glomerular Endothelial Cells via IL-6–Mediated Crosstalk

## Data Supplement

**Files in this Data Supplement:**

- Supplemental Figures 1 (PDF)
