# Supplementary material for: Podocytes Regulate Neutrophil Recruitment by Glomerular Endothelial Cells via IL-6–Mediated Crosstalk
Source: J Immunol. 2014 May 28;193(1):234–43. doi: 10.4049/jimmunol.1300229 (PMC4067868; doi:10.4049/jimmunol.1300229)
Supplement: Data Supplement [file 1300229_JI_1300229_Supplemental_Figures_1.pdf]

[1]

Supplementary Figure 1

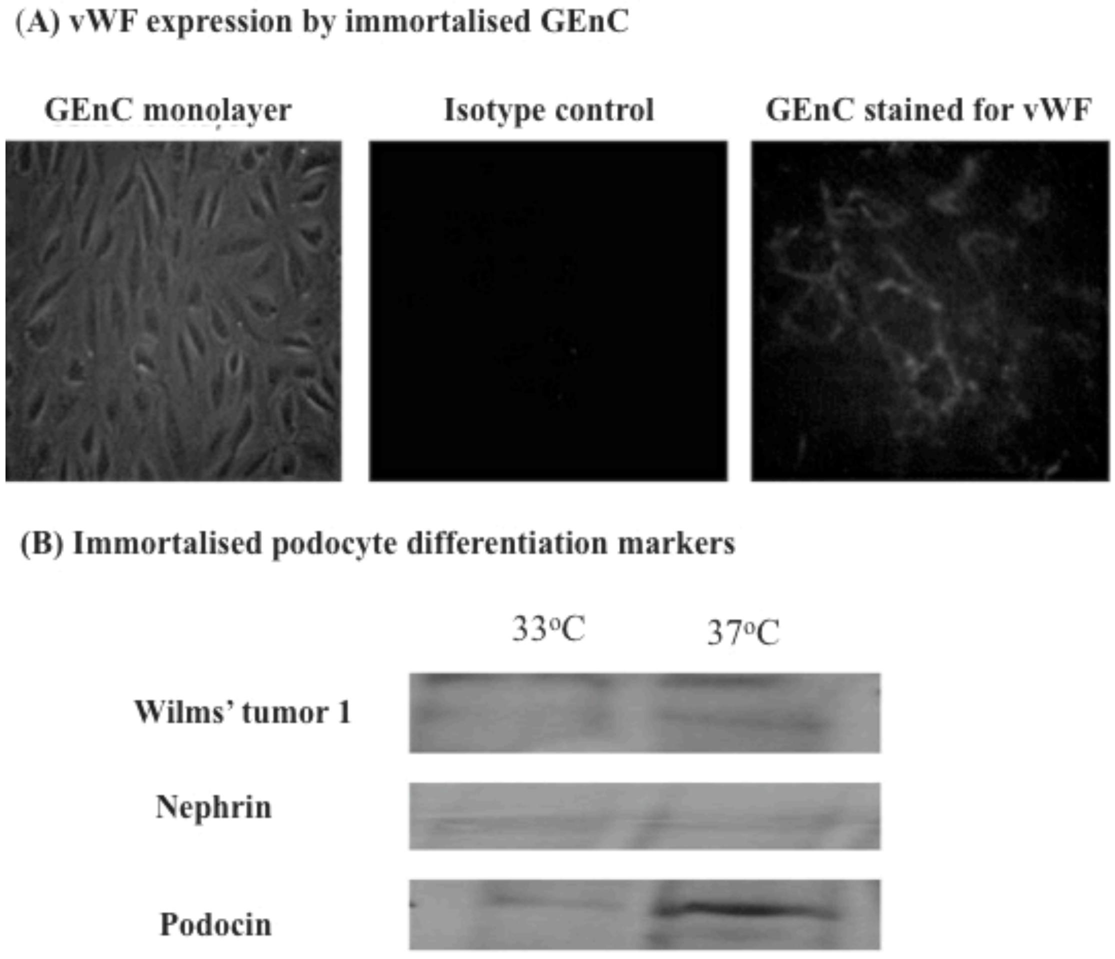

---

[1] **Supplementary Figure 1**: **Markers of GEnC and podocytes were shown expressed by differentiated immortalised cell lines.** (A) Light microscopy and immunofluorescent images of GEnC stained for von Willebrand factor (vWF) and isotype control. (B) Western blots of podocyte cell lysates from undifferentiated (33°C) and 14 days differentiated (37°C) podocytes showing markers, wilm's tumor 1 (WT-1), nephrin and podocin.

2

Supplementary Figure 2

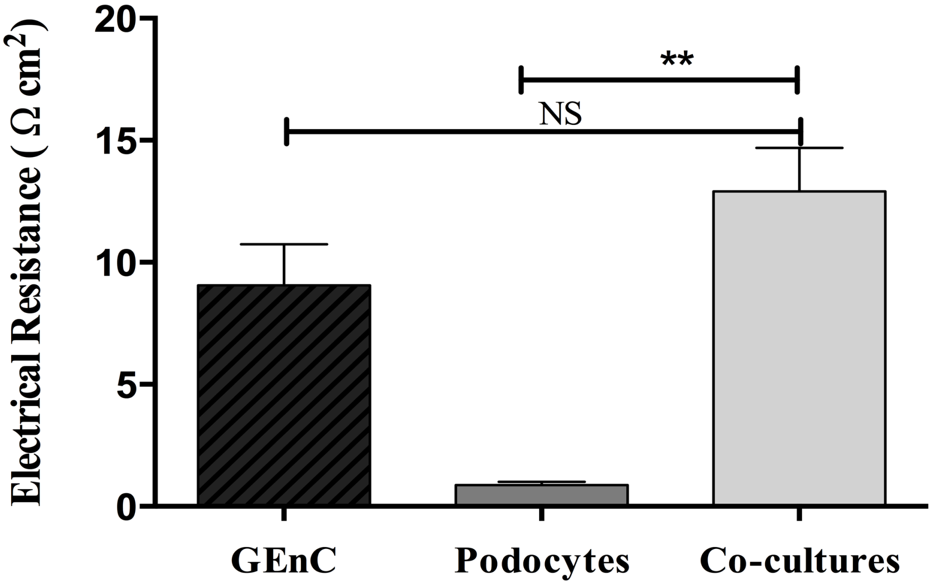

---

2 **Supplementary Figure 2:** Monolayer integrity was assessed by measuring the electrical resistance across podocyte monolayers, GEnC monolayers and co-cultured inserts. Data are mean +/- SEM, n=43. **=p<0.01 the monocultures were compared to co-cultures by Mann-Whitney test.

[3]

Supplementary Figure 3

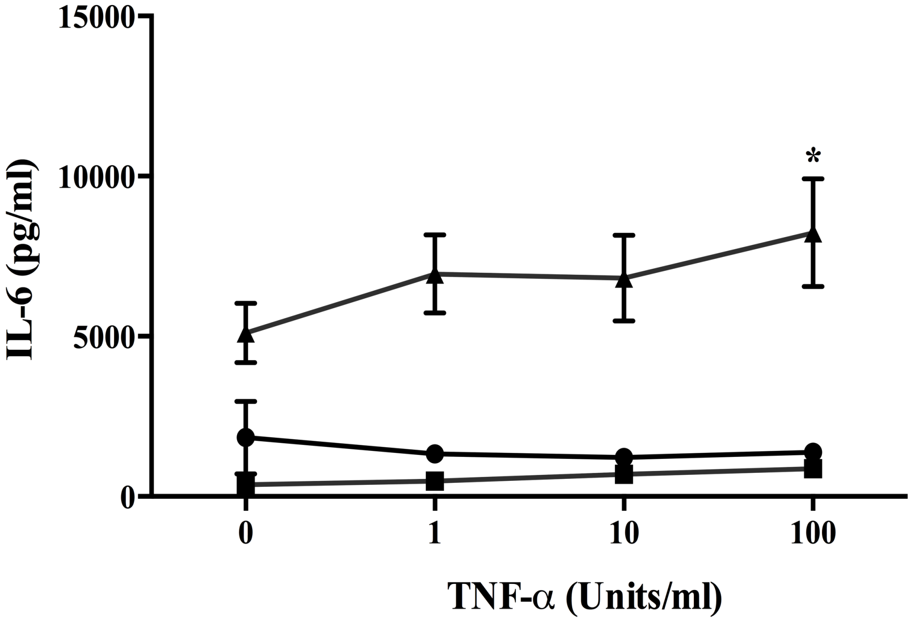

---

[3] **Supplementary Figure 3: Detection of IL-6 in culture supernatants of HUVEC and podocyte co-culture by multiplex ELISA.**

Detection of IL-6 in supernatants collected from HUVEC and podocyte co-cultures (triangles), HUVEC monocultures (squares) and podocyte monocultures (triangles) in the presence and absence of TNF-α treatment. Data are mean +/- SEM, n=4. *=p<0.05 by two way ANOVA compared between cultures and *=p<0.05 with Bonferroni post test difference in levels of IL-6.
